# Factors associated with chemical burns in Zhejiang province, China: An epidemiological study

**DOI:** 10.1186/1471-2458-11-746

**Published:** 2011-09-30

**Authors:** Yuan H Zhang, Chun M Han, Guo X Chen, Chun J Ye, Rui M Jiang, Li P Liu, Liang F Ni

**Affiliations:** 1 Zhejiang Quhua Hospital, No. 62, Wenchang Road, Quhua District, Quzhou, Zhejiang Province, 324004, China; 2 the Second Affiliated Hosptial, Zhejiang University College of Medicine, No. 88, Jiefang Road, Hangzhou, Zhejinang, 310009,China

**Keywords:** Burn prevention, burn treatment, chemical burn, epidemiology, occupational injury

## Abstract

**Background:**

Work-related burns are common among occupational injuries. Zhejiang Province is an industrial area with a high incidence of chemical burns. We aimed to survey epidemiological features of chemical burns in Zhejiang province to determine associated factors and acquire data for developing a strategy to prevent and treat chemical burns.

**Methods:**

Questionnaires were developed, reviewed and validated by experts, and sent to 25 hospitals in Zhejiang province to prospectively collect data of 492 chemical burn patients admitted during one year from Sept. 1, 2008 to Aug. 31, 2009. Questions included victims' characteristics and general condition, injury location, causes of accident, causative chemicals, total body surface area burn, concomitant injuries, employee safety training, and awareness level of protective measures. Surveys were completed for each of burn patients by burn department personnel who interviewed the hospitalized patients.

**Results:**

In this study, 417 victims (87.61%) got chemical burn at work, of which 355 victims (74.58%) worked in private or individual enterprises. Most frequent chemicals involved were hydrofluoric acid and sulfuric acid. Main causes of chemical injury accidents were inappropriate operation of equipment or handling of chemicals and absence of or failure to use effective individual protection.

**Conclusions:**

Most chemical burns are preventable occupational injuries that can be attributed to inappropriate operation of equipment or handling of chemicals, lack of employee awareness about appropriate action and lack of effective protective equipment and training. Emphasis on safety education and protection for workers may help protect workers and prevent chemical burns.

## Background

The chemical industry has a strong presence in Zhejiang Province, which is the 10^th ^largest province in China. In recent years, large chemical corporations have expanded their plants and increased production. In the wake of new joint ventures, private enterprises and sole proprietorships, chemical burn casualties are on the rise, with occasional mass-injury chemical burn accidents.

Work-related burns are a common type of occupational injury and are reported to be a leading cause of occupational injury in the US [1] and Canada [2]. Previous epidemiologic studies have analyzed the incidence and features of occupational burns in various industries and specific locations around the world [2-8]. The causes of chemical burns vary based on socioeconomic activity of a particular country or region; the etiology of burns is shown to be related to geographic location, population, types of industry and social environment [3]. Although causes and incidence vary by occupation and industry, 29% of burns requiring hospitalization are said to be work related [4]. Chemical burns after thermal injury such as fire and scalding have been reported as the second most common cause of burns in China and the majority (67.8%) of these burns occur in the work environment [5]. An epidemiologic study conducted at a Singapore burn center receiving 93% of burn cases from that area studied the demographic and clinical characteristics, causes, hospital stays and mortality of burn patients treated in a 5-year period and determined that 33.4% were occupational injuries, a decrease of 11.6% from 7 years earlier, believed due to improved prevention; investigators suggested that most injuries were preventable and that management of chemical burns needed additional scrutiny especially in terms of regulations, procedures and personal protective gear [3].

Safety interventions and prevention of workplace chemical burns are common issues in the literature. A study of work-related burn injuries in the province of Ontario, Canada compared incidence and patterns of burn injuries to those of a similar study conducted 10 years prior and found no changes, suggesting that prevention strategies applied had not been effective [2]. So, the need for the development of appropriate prevention strategies is urgent. A study of occupational health and safety (OHS) in Tanzanian hospitals concluded that providing information and training and creating awareness among employees may lead to improving health and safety among workers [6]. Another analysis of occupational burns advised that quantifying and analyzing work-related burns in various settings is essential to establishing effective safety interventions [7]. Hunt et al. also suggested that education and prevention efforts might be directed at workers in high-risk occupations and especially in the age range of 25-35, shown in their study to be most affected [8].

We conducted a prospective observational study on chemical burns using standardized data from a specific time period and geographic area of China in order to understand the incidence and nature of the increased number of chemical burns occurring in Zhejiang province, which might also contribute to broader knowledge of burn accident sources and rates, worker demographics and related workplace conditions. We followed the suggestion of previous studies that collected data may be useful in developing a strategy to prevent chemical burns and to provide a knowledge base for developing an emergency response plan for mass chemical burns. The purpose of this study was to investigate the clinical features of burn patients and circumstances of injuries in order to identify associated factors and develop a strategy for effective chemical burn prevention and treatment.

## Methods

This is a prospective observational study of standardized data from a defined period of time in a specific geographical area of China. We used a questionnaire developed by our research team and validated by an expert panel at the Zhejiang Provincial Guidance Center for Burn Treatment, a governmental organization under the of the Health Bureau of Zhejiang Province. A pilot study conducted previously on ten burn patients determined that this questionnaire (Additional files 1 and 2), with modest adjustments after the pilot study, was able to fulfill the needs of the present study. This study surveyed 527 consecutive hospitalized patients with chemical burns treated by burn surgeons in Zhejiang Province in the one-year period between September 1, 2008 and August 31, 2009. However, patients treated by other specialists such as an ophthalmologist were not included. A total of 25 hospitals with a department of burn or a burn team participated in this study. Among them, Zhejian Quhua Hospital took a leading role in the questionnaire design, and implementation, oversight, and summarization of the study. The study protocol was approved by the Ethics Committee of Zhejiang Quhua Hospital, and all participating patients provided written informed consent.

The questionnaire collected data regarding the following aspects: background information, patients' demographic characteristics, location of accident, season of accident, cause of injury, causative chemical, onsite management of wound, body part and surface area affected, concomitant injury, mass casualties, treatment and outcome, and protective and onsite first-aid equipment and its use. Burn department personnel obtained the information for the questionnaire by interviewing the hospitalized burned patients and filling out the respective answers and by reviewing patients' records during the hospitalization period.

Among 527 consecutive chemically burned patients, only 492 patients were finally retained into analysis because35 patients were excluded due to missing records. These patients were classified into two groups according to subjects' knowledge of the immediate treatment required for the wound. Among them, 263 patients (53.46%) who were aware that the wound should be immediately treated on site were classified as group 1, and 229 patients (46.54%) who did not know were classified as group 2.

### Statistical Analysis

Continuous data were summarized as median and inter quartile range (IQR: Q1, Q3) since data were not normally distributed. Differences between groups were examined by Mann-Whitney U test. Categorical variables were summarized as count and percentage, and their associations within the patients' groups were tested with Fisher's exact test. All statistical assessments were two-tailed and performed with a significance level of p < 0.05. Statistical analyses were performed by using SAS 9.1 (SAS Institute Inc., Cary, NC).

## Results and Discussion

### Patients' demographic characteristics

A total of 492 questionnaires were included in our analysis. Demographic data are shown in Table 1. There were 408 male (82.93%) and 84 female patients (17.07%), with male to female ratio of 4.9 to 1. The average age of the included patients was 38.04 ± 11.91 years old. Ages ranged from 1 to 81 years (38(30, 45)). Regarding education levels, 145 patients (29.47%) had elementary school or lower; 232 (47.15%) completed junior high school; 89 (18.09%) completed senior high school or secondary technical and vocational schools; and 26 (5.29%) had college or higher. Among 417 patients (87.60%) with occupational injures (injuries at work), 32 (6.72%) worked for state-owned enterprises, 30 (6.30%) worked for wholly foreign-owned or joint-venture enterprises, and 355 (74.58%) were employees of private enterprises and sole proprietors. Another 59 cases (12.40%) were injured from non-occupational causes. There were 16 missing values. Most burn injuries occurred in private enterprises and sole proprietorships.

**Table 1:** Demographic characteristics of two patient groups

Variable ^a^	All (N = 492)	Group 1^b ^(N = 263)	Group 2 ^b ^(N = 229)	P-value
Age (year)	38(30,45)	38(30,45)	39(30,45)	0.825
Gender				< 0.001*
Male	408(82.93%)	243(92.40%)	165(72.05%)	
Female	84(17.07%)	20(7.60%)	64(27.95%)	
Marital Status				0.838
Married	395(80.29%)	214(81.37%)	181(79.04%)	
Single	93(18.90%)	47(17.87%)	46(20.09%)	
Divorce	4(0.81%)	2(0.76%)	2(0.87%)	
Educational background				0.037*
Elementary school	145(29.47%)	86(32.70%)	59(25.76%)	
Junior high school	232(47.15%)	123(46.77%)	109(47.60%)	
Senior high school	89(18.09%)	37(14.07%)	52(22.71%)	
College/University	26(5.29%)	17(6.46%)	9(3.93%)	
Work type				0.124
Temporary	262(53.25%)	144(54.75%)	118(51.53%)	
Permanent	137(27.85%)	78(29.66%)	59(25.76%)	
Other	93(18.90%)	41(15.59%)	52(22.71%)	
Organization^†^				< 0.001*
Government owned	32(6.72%)	28(10.65%)	4(1.88%)	
Foreign enterprises	30(6.30%)	24(9.12%)	6(2.82%)	
Private enterprises	355(74.58%)	179(68.06%)	176(82.63%)	
Other	59(12.40%)	32(12.17%)	27(12.67%)	

^a ^Age was presented as median (IQR) and compared by Mann-Whitney U test; categorical variables were presented as count (percentage) and tested by Fisher's exact test

^b ^Patients were classified into two groups according to awareness of correct and immediate onsite treatment required (Group 1) or lack of awareness of immediate treatment required (Group 2)

*P < 0.05 indicated significant differences between groups

^†^There were 16 missing value.

### Comparison of subjects' demographic data

Analysis of demographic data of the two groups of patients is presented in Table 1. A significant association was shown in gender. Group 1 had fewer females than group 2 [20 (7.60%) v.s. 64(27.95%), p < 0.001]. The two patient groups were significantly different in distribution of educational background (p = 0.037). Furthermore, these patient groups were significantly different in distribution of organization (p < 0.001). However, there were no significant differences in age, marital status, and work type between patients groups.

### Accident characteristics

#### Location of accident

Most burns (321; 65.24%) occurred in the production shop of the particular industry; 5 (1.02%) occurred in the laboratory; 48 (9.76%) occurred during transportation; 69 (14.02%) occurred during handling of the chemical substance; and 49 cases (9.96%) occurred in other locations.

#### Causes of injury

Inappropriate operation of equipment or handling of chemicals, 351 cases (71.34%); facility problems such as aged parts or insufficient technology, 88 cases (17.89%); and other causes, including environmental factors, daily life accidents, attempted assault and attempted suicide etc., 53 cases (10.77%).

#### Causative chemicals

The most common causative chemicals were hydrofluoric acid and sulfuric acid. Causative chemicals are shown in Table 2.

**Table 2:** Causative substances of chemical burns

Substance	No. of cases	Percentage
Hydrofluoric acid	135	27.44
Sulfuric acid	115	23.37
Mixed acid	41	8.33
Sodium (potassium) hydroxide	33	6.71
Nitric acid	31	6.30
Hydrochloric acid	27	5.49
Lye	18	3.66
Phenol	14	2.85
Calcium oxide	11	2.24
Bromine	10	2.03
Bitumen	8	1.63
Acetic acid	7	1.42
Liquid ammonia	7	1.42
Aromatic amino and nitro compounds	6	1.22
Dimethyl sulfate	5	1.02
m-cresol	4	0.81
Yellow phosphorous	4	0.81
Others	16	3.25

#### Concomitant injury

There were 41 cases of concomitant inhalation injury, mostly resulting from irritants and volatile chemicals such as hydrofluoric acid, bromine, liquid ammonia, and hydrochloric acid. Among these, 22 were mildly injured, 14 were moderately injured, and 5 were severely injured. In addition, 10 cases of concomitant chemical poisoning were noted, mostly caused by hydrofluoric acid, followed by aromatic amino and nitro compounds. There was one case of concomitant crush injury, one case of bone fracture, and one case of craniocerebral trauma.

#### Protective and onsite first-aid equipment and its use

Only 137 out of 434 victims of occupational injuries had been given orientation training including safety aspects; 62 had received a training period of a week or shorter, and another 32 had received a training period of one month or longer. The employers of 155 cases had provided protective equipment, which was worn by merely 64 patients as instructed. Regarding knowledge about putting on such equipment, 190 patients believed that it was necessary, and another 109 thought that it was necessary but inconvenient, and yet another 132 considered it unimportant. Emergency showers were available in the workplace of 160 patients, while 271 patients did not have or were unaware of such equipment in their workplaces. First-aid stations and emergency medicine were present in the workplaces of 17 and 50 patients, respectively, while another 366 reported that neither of these was available.

#### Onsite management of wound

A total of 231 patients received massive water irrigation and 17 patients applied the wound with neutralizers following water irrigation. Most onsite water irrigation began within five minutes after the accident and lasted from 1 to 60 (16.22 ± 10.62) minutes.

#### Treatment and outcomes

Among 128 patients who received surgical treatment for burn wounds, 124 cases were given early-stage eschar excision or late-stage granulating wound debridement and skin grafting, and 4 cases were given transposition skin flaps. A total of 485 patients were discharged after healing or stabilization. Three patients transferred to another hospital. One patient was discharged against advice (the patient gave up treatment on the 13^th ^day after injury). Among the three deaths, two died of hydrofluoric acid poisoning and one of severe concomitant crush injury.

The association of clinical features of patient groups is analyzed in Table 3. Two patient groups were significantly different in causes of chemical burn, first aid treatment, and pre-job training (p < 0.001). Group 1 had less inappropriate operation rate than group 2. The majority of patients in group 1 did immediately treat the wounds on site and had received pre-job training before. Unexpectedly, compared to patients in group 2, patients in group 1 had higher burning area (p < 0.001), and suffered from more severe inhalation and chemical poison (p = 0.007 and 0.032, respectively). The above findings suggested that knowing the concept that the wound should be immediately treated on site did not reduce the damage caused by chemical burn. There is no difference in prognoses status between two patient groups (p = 0.078).

**Table 3:** Clinical features of all patients classified by patient groups

Variable ^a^	All (N = 492)	Group 1^b ^(N = 263)	Group 2 ^b ^(N = 229)	P-value
Cause of chemical burns				< 0.001*
Facility problem	88(17.89%)	74(28.14%)	14(6.11%)	
Inappropriate operation/handling	351(71.34%)	161(61.22%)	190(82.97%)	
Other	53(10.77%)	28(10.64%)	25(10.92%)	
First aid treatment				< 0.001*
Without any treatment	241(48.98%)	13(4.94%)	228(99.56%)	
Flush with running water	231(46.95%)	230(87.45%)	1(0.44%)	
Using counteractive agent	17(3.46%)	17(6.47%)	0(0%)	
Other	3(0.61%)	3(1.14%)	0(0%)	
				
Total body surface area burned (%)	4(1.5,8)	5(3,10)	2(1,7)	< 0.001*
				
Inhalation				0.007*
No	450(91.65%)	230(87.78%)	220(96.07%)	
Slight	22(4.48%)	18(6.87%)	4(1.75%)	
Moderate	14(2.85%)	10(3.82%)	4(1.75%)	
Severe	5(1.02%)	4(1.53%)	1(0.43%)	
Chemical poisoning				0.032*
No	482(97.97%)	254(96.58%)	228(99.56%)	
Slight	4(0.81%)	4(1.52%)	0(0%)	
Moderate	4(0.81%)	4(1.52%)	0(0%)	
Severe	2(0.41%)	1(0.38%)	1(0.44%)	
Pre-job training^**†**^				< 0.001*
Yes	137(31.64%)	115(49.78%)	22(10.89%)	
No	296(68.36%)	116(50.22%)	180(89.11%)	
Prognoses				0.078
Cured	401(81.51%)	207(78.71%)	194(84.72%)	
Improvement	84(17.07%)	54(20.53%)	30(13.10%)	
Hospital transfer	3(0.61%)	1(0.38%)	2(0.87%)	
Discharged against advice	1(0.20%)	0(0%)	1(0.44%)	
Death	3(0.61%)	1(0.38%)	2(0.87%)	

^a ^Burned area was presented as median (IQR) and compared by Mann-Whitney U test; categorical variables were presented as count (percentage) and tested by Fisher's exact test

^b ^Patients were classified into two groups according to awareness of correct immediate onsite treatment required (Group 1) or lack of awareness of immediate treatment required (Group 2)

*P < 0.05 indicated significant differences between groups

^**†**^Fifty nine patients who were not injured at work were excluded from this analysis.

In our survey, inappropriate operation or handling of chemicals, lack of employee awareness about appropriate action and a lack of effective personal protective equipment were key factors contributing to chemical burns. We found that the workplaces of private enterprises and sole proprietors had a greater share of occupational injuries than larger industrial companies. This may be due to the more recent and rapid development of private business in Zhejiang where middle- to small-sized private chemical companies are abundant throughout the province. It may also be the result of the lower education levels of employees in private enterprises and the higher turnover rate, which means that most employees were without orientation training, operational proficiency and awareness of safety issues, coupled with a lack of effective onsite first-aid. The companies also appeared to be suffering from failure of management since 62.2% of the burned patients were working in workplaces deprived of any protective equipment. Even when such equipment was provided, only 42.0% of patients put on the protective gear as instructed, indicating lack of safety education and training in procedures. These results suggest that the effective route to chemical burn prevention is through enforcing safety education, improving awareness and supervision of all personnel, and providing personal protection for all employees.

It is noteworthy that a certain number of chemical burns occurred in non-chemical industries. Protection was generally inadequate in these companies due to a lack of awareness at the managerial level down to the common first-line workers of the hazards related to the chemicals being used. Prior to the present study, no systematic investigation was made of the occurrence of burn accidents and events. The potential hazards of low-grade hydrofluoric acid (about 5% concentration or 2.5 mol/L), the main component of the rust remover Qu-xiu-ling (triadimefon), which is readily available in the market, are often ignored because the agent causes little skin discomfort at first contact but results in chemical burns 2-3 hours later if left untreated [9]. The product label of Qu-xiu-ling failed to include the ingredients on the outer packaging. Although there were notices requiring users to wear rubber gloves, the warning was insufficient and unnoticeable. Therefore, we recommend that in addition to the enforcement of safety education, there should be prominent warning signs on the outer packages of potentially dangerous chemical products in order to prevent similar events from occurring. This could perhaps be influenced by the purchasing departments of industries that use the products.

Among all substances contributing to chemical burns, hydrofluoric acid caused the largest share of casualties, likely due to the high concentration of the fluorochemical industry in Zhejiang. Besides the presence of large fluorochemical plants that are engaged in inorganic or organic fluorine production, the province has seen a rapid growth of small- and middle-sized fluorochemical companies in recent years. These plants, large or small, either produce hydrofluoric acid or use hydrofluoric acid as a raw material. There is a potential for chemical burns during the production, loading, and transportation of hydrofluoric acid. Furthermore, since low-grade hydrofluoric acid is used as a cleaning agent in metal casting, glass processing, and electronics industries, it results in a higher incidence of burns. Burns caused by hydrofluoric acid are fairly uncommon but when they do occur they can be severe and sometimes fatal [10]. Hydrofluoric acid has been responsible for mass casualties. It is also notable that a medium or small area of hydrofluoric acid burn can generate severe chemical poisoning [10]. Following a hydrofluoric acid burn, fluoride rapidly enters blood circulation through the skin and concentration peaks within an hour [11]. With swift development, fluoride poisoning can lead to death in a short period of time due to life-threatening electrolyte disturbances, even as a result of small, highly concentrated acid burns [4]. Throughout the study period, the two deaths from chemical poisoning were caused by hydrofluoric acid burns and these patients died within eight hours following the accident. Hatzifotis et al. presented a management strategy for hydrofluoric acid burns that included the application of calcium gluconate with dimethyl sulphoxide (DMSO) in surgical jelly for the potentially fatal injury caused by cutaneous exposure to hydrofluoric acid [10]. This method could easily be incorporated into industry procedures for emergency care. As in most occupational burn cases, treatment should commence immediately after at least 30 minutes of lavage with water. Clinical management of hydrofluoric acid burns calls for immediate washing of the burned area with water and external application of calcium as described above [10]. For hypocalcemia patients, calcium gluconate should be injected intravenously, depending on the individual patient's blood calcium levels. Arterial delivery of calcium gluconate is also possible, which allows the drug to combine with fluoride ions in the wounds, a method generally applied to hydrofluoric acid burns on the extremities (e.g., fingers and toes) [9,12] or to burns on the head and face [13]. Because of the special and dangerous nature of hydrofluoric acid burns, Kirkpatrick and Burd (1995) developed an algorithmic approach to treatment of these burns and it remains a reference for clinicians [14]. In our study, surviving patients with deep burns from hydrofluoric acid were left with scars after healing, but with no other sequelae. However, when treatment of hydrofluoric acid burns to the fingers was delayed more than 48 hours, the distal end of the fingers would show signs of necrosis, turn black, and would become shortened.

After contact with the human body, chemicals not only produce immediate harm through inflammatory response, but they also cause progressive damage leading to epidermal and dermal lesions [15] and major burns affecting a significant body surface area may lead to compromise of the circulation and air exchange, fluid and electrolyte imbalance and systemic shock and sepsis [16]. Some chemicals such as hydrofluoric acid can be absorbed and produce systemic chemical poisoning. The delivery of effective onsite management in a timely fashion is a direct determinant of the degree and prognosis of damage. Our survey showed that no skin graft surgery was needed for all patients whose wounds had been immediately irrigated with massive amounts of water for 30 minutes or more, and all those requiring skin graft or transposition skin flaps had received inadequate onsite wound management. In spite of varying substances and different causes of damage, massive water irrigation is the most convenient and effective means of onsite first-aid, for it not only prevents further harm by washing out the chemical, but it serves as a cooling agent by taking away the heat. Provided there is no life-threatening concomitant injury, water irrigation should be initiated as early as possible and continue for a minimum of 30 minutes [17]. We also found that about half of the patients were not aware of the correct onsite management of chemical burns. Some patients, and even the attending medical staff, had a clear misunderstanding that water plus concentrated sulfuric acid produces heat, hence aggravating the damage. There is an urgent need to promote knowledge about correct chemical burn first-aid so that the correct action can be taken early onsite instead of waiting for treatment by medical professionals.

Among work-related burns, those of the upper extremities occur most often in our study and others and it is the cause that directs the course of action [4]. For example, thermal or heat burns must be assessed for depth and for systemic effects. The deepest partial-thickness burns and more severe burns with pulmonary effects or shock require evaluation by burn specialists outside the workplace. Chemical burns, as we've noted, require irrigation and identification of the causative chemical; however, the causative chemical may indicate that specific topical applications are needed before water lavage such as for phenols and metal fragments [4].

Onsite protection in industry for exposure to skin irritants first requires physical protective countermeasures such as the wearing of protective gear for operating equipment or handling chemicals; it may also require availability of appropriate pharmacologic topical treatment to prevent the development of burns or to provide an immediate antidote if harmful exposure occurs, either of which may help avoid complications and later surgical treatment of burns [15]. In a statewide, cross-sectional study of all workplace burns during one year, most of the injuries were to the upper extremities and head and were caused by either exposure to caustic chemicals or hot objects or substances; investigators concluded that availability and use of protective gear for the upper extremities and head could prevent a significant number of this type of injury and that education and prevention programs directed to high-risk workers was the best management approach [8]. Burn injuries outside of the workplace have been declining, largely as a result of safer homes (e.g., smoke detectors, home construction measures), burn education in schools and focused efforts to reduce home fires [8]. Prevention of occupational burns clearly requires a similar focus.

Our study is limited by the use of a new Chinese questionnaire designed by the authors and not previously tested or validated. This study also focused exclusively on patients with chemical burns and did not survey all industries or all workers for work-related injuries. Future study must be more comprehensive, including all industries and all workers in a given population.

## Conclusions

In conclusion, our results strongly suggest that preventing occupational burn injuries due to inappropriate operation of equipment or handling of chemicals lies in increasing employee awareness about appropriate action and ensuring the provision of effective protective equipment and training. Emphasizing safety education, physical protection for workers and adequate information about emergency measures may directly benefit workers and reduce the occurrence of chemical burns.

## Competing interests

The authors declare that they have no competing interests.

## Authors' contributions

YHZ drafted the manuscript. CJ Ye, RMJ, LPL and LFN participated in the design of the study and performed the statistical analysis. YHZ, CMH and GXC conceived of the study, and participated in its design and coordination and helped to draft the manuscript. All authors read and approved the final manuscript.

## Pre-publication history

The pre-publication history for this paper can be accessed here:

http://www.biomedcentral.com/1471-2458/11/746/prepub

## Supplementary Material

Additional file 1 **Questionnaire for chemical burn patients in chinese**. A description of burn patients ' background information, description of injury, onsite wound management, training on related know-how and use of protective gears, availability and use of emergency shower and first-aid facilities in Chinese. Click here for file

Additional file 2 **Questionnaire for chemical burn patients in English**. A description of burn patients ' background information, description of injury, onsite wound management, training on related know-how and use of protective gears, availability and use of emergency shower and first-aid facilities in English. Click here for file
