# Supplementary material for: Factors associated with chemical burns in Zhejiang province, China: An epidemiological study
Source: BMC Public Health. 2011 Sep 30;11:746. doi: 10.1186/1471-2458-11-746 (PMC3196712; doi:10.1186/1471-2458-11-746)
Supplement: Additional file 1 — Questionnaire for chemical burn patients in chinese. A description of burn patients ' background information, description of injury, onsite wound management, training on related know-how and use of protective gears, availability and use of emergency shower and first-aid facilities in Chinese. [file 1471-2458-11-746-S1.DOC]

**浙江省化学烧伤病人调查表**

一、就诊医院————————————————— 住院号——————————

二、一般情况：

1.姓名——————  2.性别：男（ ） 女（ ） 3.年龄：———— 4.婚姻：已（ ） 未（ ）离（ ）

5.文化程度：小学及以下（ ） 初中（ ） 高中（含技校、中专）（ ） 大专及以上（ ）

6.工种：临时工（ ） 正式工（ ）其它：—————— 7.从事受伤工种工龄：———年———月———日

8.受伤地点：生产车间（ ） 实验室（ ） 运输途中（ ） 使用过程中 其它——————

9.单位名称：———————————— 单位性质：国营（ ） 外企或合资（ ） 民营（ ） 其它————

10.受伤原因：（1）设备原因（老化或工艺不成熟等）（ ） （2） 操作不当（ ）

（3）自杀 （4）别人攻击（ ） （5）其它————————

11.烧伤日期：———年———月———日 12. 入院时间：伤后———天———小时 13. 住院时间—————天

三、受伤情况：

1.致伤的化学物质名称_—————————————、_

2.烧伤面积——————BSA% 其中： 浅Ⅱ度————— 深Ⅱ度————— Ⅲ度————— Ⅳ度—————

3.烧伤部位：头面、颈、前躯、后躯、上臂、前臂、手、臀、会阴、下肢

4.合并伤：（1）吸入性损伤：无（ ）轻度（ ） 中度（ ） 重度（ ）

（2）化学中毒： 无（ ） 轻度（ ） 中度（ ） 重度（ ） （3）其它—————————

5.手术治疗：是（ ） 否（ ）

（1）手术名称——————————————— （2）手术时间：伤后———天———小时

6.治疗转归：治愈（ ） 好转（ ） 转院（ ） 自动出院（ ） 死亡（ ）

四、现场处理情况：

1.是否知道创面需立即现场处理：是（ ） 否（ ）2.是否及时进行了现场处理：是（ ） 否（ ）

3.现场处理方法：（1）大量水冲洗（ ） 水冲洗时间：———分钟 开始时间：伤后———分钟

（2）使用中和剂（ ） （3）其它———————

五、相关知识的培训和防护用品的使用情况：

1.岗前是否接受过培训：是（ ）否（ ） 培训时间：———月———天

2.厂方是否配备防护用品：是（ ）否（ ）不清楚 ( )

3.受伤时是否穿戴防护用品：是（ ） 否（ ） 穿戴了但不符合要求（ ）

4.伤者对穿戴防护用品的认识，认为：有必要（ ）有必要但不方便（ ） 无所谓（ ）

六、急冲设备和急救用品的配备和使用情况：

1.现场是否有急冲设备：是（ ）否（ ） 不清楚 ( ) 2.设备是否运行良好：是（ ）否（ ）

3. 急冲设备使用是否方便：是（ ）否（ ）不清楚 ( )

4.厂方急救用品的配备情况：

（1）设有急救站（ ）、（2）配备急救药品（ ）、（3）无配备（ ）

填表日期—————————— 填表人——————————
