# Supplementary material for: Factors associated with chemical burns in Zhejiang province, China: An epidemiological study
Source: BMC Public Health. 2011 Sep 30;11:746. doi: 10.1186/1471-2458-11-746 (PMC3196712; doi:10.1186/1471-2458-11-746)
Supplement: Additional file 2 — Questionnaire for chemical burn patients in English. A description of burn patients ' background information, description of injury, onsite wound management, training on related know-how and use of protective gears, availability and use of emergency shower and first-aid facilities in English. [file 1471-2458-11-746-S2.DOC]

**Questionnaire for chemical burn patients in Zhejiang**

I. Hospital————————————————— Admission No. —————————————————

II. Background information:

1. Name —————— 2. Gender: Male ( ) Female ( ) 3. Age: ————

4. Marriage status: Married ( ) Unmarried ( ) Divorced ( )

5. Education: Elementary school and below ( ) Junior high ( ) Senior high (including technical and secondary vocational schools) College and higher ( )

6. Employment: Part-time ( ) Full-time ( ) Others: ——————

7. Length of employment till the time of injury: ———years———months———days

8. Location of injury: production shop ( ) laboratory ( ) transportation ( ) during handling of the substance ( ) Others ——————

9. Name of the employer: ———————————— Employer affiliation: State-owned ( ) Foreign-owned or joint venture ( ) Private ( ) Others ——————

10. Cause of injury: (1) Equipment problems (such as aged parts or insufficient technique) ( ) (2) Inappropriate operation ( ) (3) Suicide ( ) (4) Intended assaults ( ) (5) Others ——————

11. Date of burn injury: ———year———month———day

12. Time of admission: ———days———hours after injury

13. Length of hospital stay: ———days

III. Description of injury

1. Name of the causative substance —————————————

2. Area of burn —————— BSA%, among which superficial II-degree burn —————, deep II-degree burn —————, III-degree burn —————, and IV-degree burn —————.

3. Injured body parts: Head and face, neck, front trunk, rear trunk, arm, forearm, hand, hip, perineum, lower extremity.

4. Concomitant injury: (1) Inhalation injury: none ( ) mild ( ) moderate ( ) severe ( )

(2) Chemical poisoning: none ( ) mild ( ) moderate ( ) severe ( )

5. Surgical treatment: Yes ( ) No ( )

6. Outcome: Healed ( ) Improved ( ) Transferred ( ) Discharged against advice ( ) Death ( )

IV. Onsite wound management:

1. Was the patient aware that the wound should be immediately treated onsite? Yes ( ) No ( )

2. Was the wound immediately treated onsite? Yes ( ) No ( )

3. Management method: (1) Irrigate with massive water ( ), irrigation time: ——— minutes. Irrigation started at ——— minutes after injury.

(2) Apply neutralizers ( ) (3) Others ———————

V. Training on related know-how and use of protective gears

1. Had the patient received orientation training before work: Yes ( ) No ( ) training time: ———months———days

2. Did the workplace provide protective gears? Yes ( ) No ( ) Don’t know ( )

3. Was the patient wearing protective gears at the time of injury? Yes ( ) No ( ) Yes, but failed to meet the requirements ( )

4. The patient thought protective gears were: Necessary ( ) Necessary but inconvenient ( ) Unimportant ( )

VI. Availability and use of emergency shower and first-aid facilities

1. Was onsite emergency shower available? Yes ( ) No ( ) Don’t know ( )

2. Was the equipment functioning? Yes ( ) No ( )

3. Was the equipment easy to use? Yes ( ) No ( )

4. Availability of first-aid facilities:

(1) First-aid station was established ( ), (2) emergency medicine could be found ( ), (3) neither was available ( )

Date of filling the form —————————— Person filling the form ——————————
